# Enough is enough: Alcohol‐related occupational violence and aggression in emergency departments in Australia and New Zealand

**DOI:** 10.1111/1742-6723.70021

**Published:** 2025-03-07

**Authors:** Diana Egerton‐Warburton, Jolene Lim, Dinesh Seiji Seneviratne, Sue Bumpstead, Laura R Joyce, Lisa Kuhn, Katie Moore, Drew B Richardson, Robert Lee, Daniel M Fatovich

**Affiliations:** ^1^ School of Clinical Science at Monash Health Monash University, Monash Medical Centre Melbourne Victoria Australia; ^2^ Policy, Research and Partnerships Department Australasian College for Emergency Medicine Melbourne Victoria Australia; ^3^ Peninsula Health Melbourne Victoria Australia; ^4^ Monash Emergency Research Collaborative Monash Health Melbourne Victoria Australia; ^5^ Faculty of Medicine, Nursing and Health Sciences Monash University Melbourne Victoria Australia; ^6^ Department of Surgery and Critical Care University of Otago Christchurch New Zealand; ^7^ Emergency Department Te Whatu Ora Waitaha Canterbury Christchurch New Zealand; ^8^ Australian Catholic University Melbourne Victoria Australia; ^9^ Monash Emergency Research Collaborative, Monash Health, Emergency Department Monash Medical Centre Melbourne Victoria Australia; ^10^ Australasian College for Emergency Medicine Melbourne Victoria Australia; ^11^ Australian National University Medical School Canberra Australian Capital Territory Australia; ^12^ Emergency Medicine Royal Perth Hospital, University of Western Australia Perth Western Australia Australia; ^13^ Centre for Clinical Research in Emergency Medicine Harry Perkins Institute of Medical Research Melbourne Victoria Australia

**Keywords:** alcohol, COVID, occupational violence, staff

## Abstract

**Objective:**

To determine the extent of alcohol‐related violence in EDs throughout Australia and New Zealand and the impact this has had on ED staff.

**Methods:**

A mixed methods, cross‐sectional, online survey of ED staff working in Australia and New Zealand conducted between 1 August and 11 September 2022 measuring the frequency of physical or verbal alcohol‐related aggression from patients and their relatives/carers; changes to the frequency of alcohol‐related occupational violence over the preceding 5 years; the impact of COVID‐19 on these presentations; and the perceived impact on ED function and staff well‐being.

**Results:**

A total of 1284 ED staff responded, with almost all (97.9%) reporting having experienced verbal aggression and 92.7% experienced physical aggression from alcohol‐affected patients at some point over the preceding 12 months. Alcohol‐related presentations were significantly associated with negatively impacting patient wait times (86.1%), the care of other patients (87.5%) and other patients in the waiting room (94.6%). A large majority of ED staff also noted that these presentations negatively impacted staff wellness (82.4%), workload (93.1%) and job satisfaction (78.9%). Most (68.2%) believed that the issue of alcohol‐related violence had worsened over the preceding 5 years and 46.7% believed that COVID‐19 specifically has worsened the incidence of alcohol‐related violence in the ED.

**Conclusion:**

Alcohol‐related occupational violence and aggression is experienced by almost all ED staff and the prevalence is perceived to be increasing. It results in negative impacts on both staff well‐being, the care of other patients and ED function.

## Graphical abstract

Levels of alcohol‐related occupational violence and aggression (OVA) in EDs remain unacceptably high: two‐thirds of ED staff surveyed believed alcohol‐related OVA has worsened over the last 5 years; almost all had experienced verbal aggression; and almost half suffered physical violence weekly or monthly. Evidence‐based policies for alcohol‐related patient management are required.

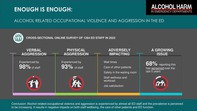

**Key findings**

- Levels of alcohol‐related OVA in EDs remain unacceptably high: two‐thirds of ED staff surveyed believed alcohol‐related OVA has worsened over the preceding 5 years; almost all had experienced verbal aggression; and almost half suffered physical violence weekly or monthly.
- Evidence‐based policies for alcohol‐related patient management are required, as is routine collection of alcohol‐related data in EDs.
- The presence of 24/7 security staff to protect ED staff must also be urgently addressed.

## Background

ED staff frequently experience verbal and physical aggression. There are multifactorial patient causes including cognitive impairment, medical conditions and trauma, as well as environmental and system issues such as overcrowding.^1^ Studies have identified alcohol use as a significant risk factor for occupational violence and aggression (OVA) faced by ED staff.^2^, ^3^ The misuse of alcohol is common and alcohol‐related presentations contribute a large portion of ED patient presentations. Almost one in 10 ED presentations are alcohol‐related with a 2014 study indicating this equates to more than half a million people per year.^4^ This increases to one in seven at peak alcohol consumption times.^5^ As a result, ED staff are at the frontline when it comes to managing alcohol‐related presentations and their consequences.

In the ED setting, alcohol use is associated with a disproportionately high use of security measures including physical, chemical or mechanical restraints.^6^ OVA also puts additional demand on staff, including the need for increased security personnel. The Department of Health identifies a case study of a health service review of security in 2017 which identified an increase in time spent on OVA responses, a process that requires security staff, as well as medical and nursing to attend. In instances when physical restraint was required, it was noted that four members of security were needed to undertake this safely.^7^

A 2014 survey demonstrated that the large majority of ED staff across Australia and New Zealand had experienced at least one episode of physical (92%) and/or verbal (98%) aggression due to alcohol‐related presentations over a period of 12 months.^8^ Survey respondents noted that alcohol‐related presentations not only posed issues to the effective functioning of EDs by increasing wait times, and affecting care of other patients, but also had negative impacts on staff well‐being and job satisfaction.^8^ Participants in the study highlighted incidents wherein alcohol affected patients required additional resources including beds and staff to manage unsafe behaviours leading to delays in investigations and care for other patients.

The COVID‐19 pandemic placed additional strain on ED clinicians,^9^ but it is unknown what effect it had on alcohol‐related OVA. While self‐reported alcohol consumption data had inconsistent results, alcohol sales data indicated increased consumption during the pandemic.^10^, ^11^ An increase in OVA during the pandemic has been reported in other service industries.^12^

Screening, brief intervention and referral to treatment (SBIRT) is a three‐step approach in identifying individuals with or at risk of substance use issues and providing early intervention. It may have utility as a countermeasure against alcohol‐related OVA by reducing the prevalence of alcohol use in the community. However, further insight into its use practically and the extent of its implementation in the ED setting is necessary.

The aim of the present study was to determine the frequency of alcohol‐related OVA experienced by Australian and New Zealand ED staff over a 12‐month period and determine its effects on staff well‐being, other patients, and ED function. A secondary aim was to investigate the perceived impact of the COVID‐19 pandemic, perceptions of change over the preceding 5 years and the frequency of screen and brief interventions in the ED.

## Methods

### Study design and participants

The present study utilised a mixed‐method, cross‐sectional survey that was based upon our previously published survey.^8^ Definitions for verbal and physical aggression were taken from the Medicine in Australia: Balancing Employment and Life (MABEL) Longitudinal Survey (http:/mabel.org.au/) (Appendix S1). An expert working group composed of an interdisciplinary team involving nursing staff, emergency physicians, academic researchers and policy leaders with expertise in alcohol and/or drug research developed several additional questions to address the changes since the onset of the COVID‐19 pandemic and these were pilot tested.

The online survey was conducted throughout a six‐week period between 1 August 2022 and 11 September 2022. The survey targeted all ED staff who were either currently working or had worked at a public or private ED within Australia or New Zealand within the past 12 months. The present study included not only doctors and nurses but also those in other roles within the ED including, but not limited to, cleaners, pharmacists, radiologists and security guards. Participation was voluntary and anonymity was maintained, with data de‐identified.

A survey link was distributed to members by the ACEM, College of Emergency Nursing Australasia (CENA), Australian College of Emergency Nursing (ACEN) and College of Emergency Nurses NZ (CENNZ). Additionally, it was distributed to Directors of Emergency Medicine and Nurse Unit Managers in ACEM‐accredited EDs in Australia and New Zealand, who were encouraged to forward the survey to all staff (clinical and non‐clinical). The study was promoted through material distributed by ACEM and *via* several social media channels. Due to the inclusive and widely distributed nature of our study, it was not possible to calculate the denominator of staff that received the survey and therefore the response rate.

### Statistical analysis

Survey responses were analysed using descriptive analysis. The associated 95% confidence intervals (CI) were calculated based on the ‘Wilson’ score interval method using EpiTools.^13^ When analysing Likert scale data, responses were combined including ‘negative’ and ‘very negative’ responses. Qualitative data were analysed independently by two researchers, categorised according to thematic keywords derived from the free‐text responses, and then summarised into major themes by the frequency distribution method. Surveys were excluded from the analysis if the respondents had not worked in the ED in the preceding 12 months or if there were incomplete or duplicate responses. Duplicates were determined by reviewing IP addresses and demographic data.

### Survey themes

The survey was composed of five sections. Questions pertained to participant demographics, their perception on how alcohol‐related presentations affected aspects of the ED, experiences of alcohol‐related violence in the ED, and screening and brief interventions in their ED. In addition, participants were afforded the opportunity to provide comments including how alcohol‐related presentations made them feel about their job, the perceived impact of these presentations on other patients in the ED and the day‐to‐day functioning of the ED.

### Ethics approval

The Monash Health Human Research Ethics Committee approved the study (RES‐22‐0000‐193L – 85365). It was considered a low‐risk study. Consent was implied through completion of the survey.

## Results

A total of 1407 staff at ED sites across Australia and New Zealand responded to the survey; 123 responses were excluded from the analysis (21 ineligible due to having not worked in an ED in the previous 12 months, one duplicate and 101 incomplete responses). The remaining 1284 where included for analysis and their demographic characteristics are described in Table 1.

**TABLE 1:** Primary workplace and demographic characteristics of survey respondents (*n* = 1284)

Workplace and demographic descriptors	*n*	%
Sex		
Female	852	66.4%
Male	416	32.4%
Prefer not to say	13	1.0%
Prefer to self‐describe	3	0.2%
ED staff role		
ED nurse	496	38.6%
EM physician/specialist	434	33.8%
EM registrar	218	17.0%
Medical officer	39	3.0%
Other†	97	7.6%
Years of working in ED(s)		
<1	58	4.5%
1–5 years	309	24.1%
6–10 years	293	22.8%
11–20 years	362	28.2%
21–30 years	188	14.6%
>30 years	74	5.8%
Primary workplace region		
Australia	1015	79.0%
ACT	43	3.3%
NSW	233	18.1%
NT	44	3.4%
QLD	208	16.2%
SA	68	5.3%
TAS	20	1.6%
VIC	301	23.4%
WA	98	7.6%
Aotearoa New Zealand	269	21.0%
Primary hospital setting		
Public	1270	98.9%
Private‡	14	1.1%
Primary workplace remoteness		
Major city	795	61.9%
Regional	451	35.1%
Remote	38	3.0%
Total respondents	1284	100.0%

† Other ED staff included the clerical staff/administrator (*n* = 41); mental health worker (*n* = 15); allied health professionals, for example, physiotherapist, pharmacist, occupational therapist (*n* = 11); social workers (*n* = 8), alcohol/other drug support worker (*n* = 6); security guard (*n* = 5); and other staff such as orderly, ED liaison officer, cleaner, etc. (*n* = 11).

‡ Private: responses from physician/specialist (*n* = 9), registrar (*n* = 1), nurse (*n* = 3) and clerical staff/admin (*n* = 1).

Verbal alcohol‐related OVA was experienced by 97.9% of respondents during the 12‐month period. Over the same period, 92.7% reported experiencing physical aggression. Table 2 provides a breakdown of how frequently respondents experienced verbal and physical aggression from both patients as well as relatives or carers.

**TABLE 2:** Proportion of respondents who reported alcohol‐related aggression in the preceding 12 months (*n* = 1240)

Frequency of alcohol‐related aggression experienced in the ED within the preceding 12 months					
	Frequently (once or more times per week)	Often (few times per month)	Occasionally (few times per 6 months)	Infrequently (few times per 12 months)	Not at all
Verbal aggression from a patient	35.6%	34.8%	19.6%	7.8%	2.1%
Physical aggression from a patient	17.6%	25.9%	29.3%	20.0%	7.3%
Verbal aggression from a relative or carer	8.5%	20.3%	27.6%	29.7%	14.0%
Physical aggression from a relative or carer	5.4%	10.2%	22.6%	36.9%	24.8%

Overall, 87.3% of respondents felt unsafe due to an alcohol‐affected patient or carer in their ED. Respondents who identified as female (89.1%; 95% CI: 86.7–91.0) were more likely than those who identified as male (83.9%; 95% CI: 80.0–87.2) to report feeling unsafe. Nursing staff (94.1%; 95% CI: 91.7–95.9) were more likely than doctors (83.7%; 95% CI: 80.7–86.3) to report feeling unsafe due to the presence of an alcohol‐affected patient in their ED.

Over the preceding 5 years, 68.2% of respondents reported that the frequency of alcohol‐related violence was a little or a lot worse and 26.5% reported no change. Nursing staff (78.5%; 95% CI: 74.6–81.9) were significantly more likely than doctors (60.9%; 95% CI: 57.1–64.5) to report that alcohol‐related violence incidents had worsened in the preceding 5 years.

**Figure 1:**
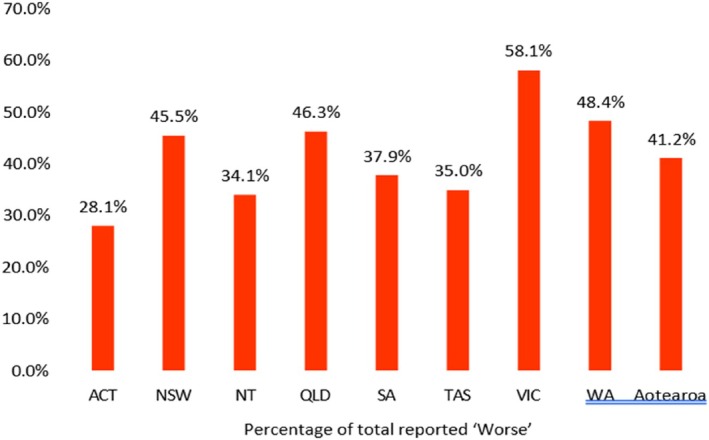
Perceived impact of COVID‐19 on incidents of alcohol‐related OVA in their EDs, by region. Error bar shows the lower and upper limit of 95% confidence interval.

COVID‐19 was perceived by 46.7% as worsening incidents of alcohol‐related violence in EDs a little or a lot, while 42.5% reported no perceived change in incidents of alcohol‐related violence in EDs and 10.8% an improvement. Nursing staff (51.7%; 95% CI: 47.2–56.1) more often reported that alcohol‐related violence incidents had become worse due to the impact of COVID‐19, compared to doctors (41.7%; 95% CI: 38.0–45.5). Figure 1 demonstrates the perceived impact based on region. Victoria remains the clear outlier, as the only region with a majority of respondents reporting that COVID‐19 has worsened alcohol‐related OVA.

**Figure 2:**
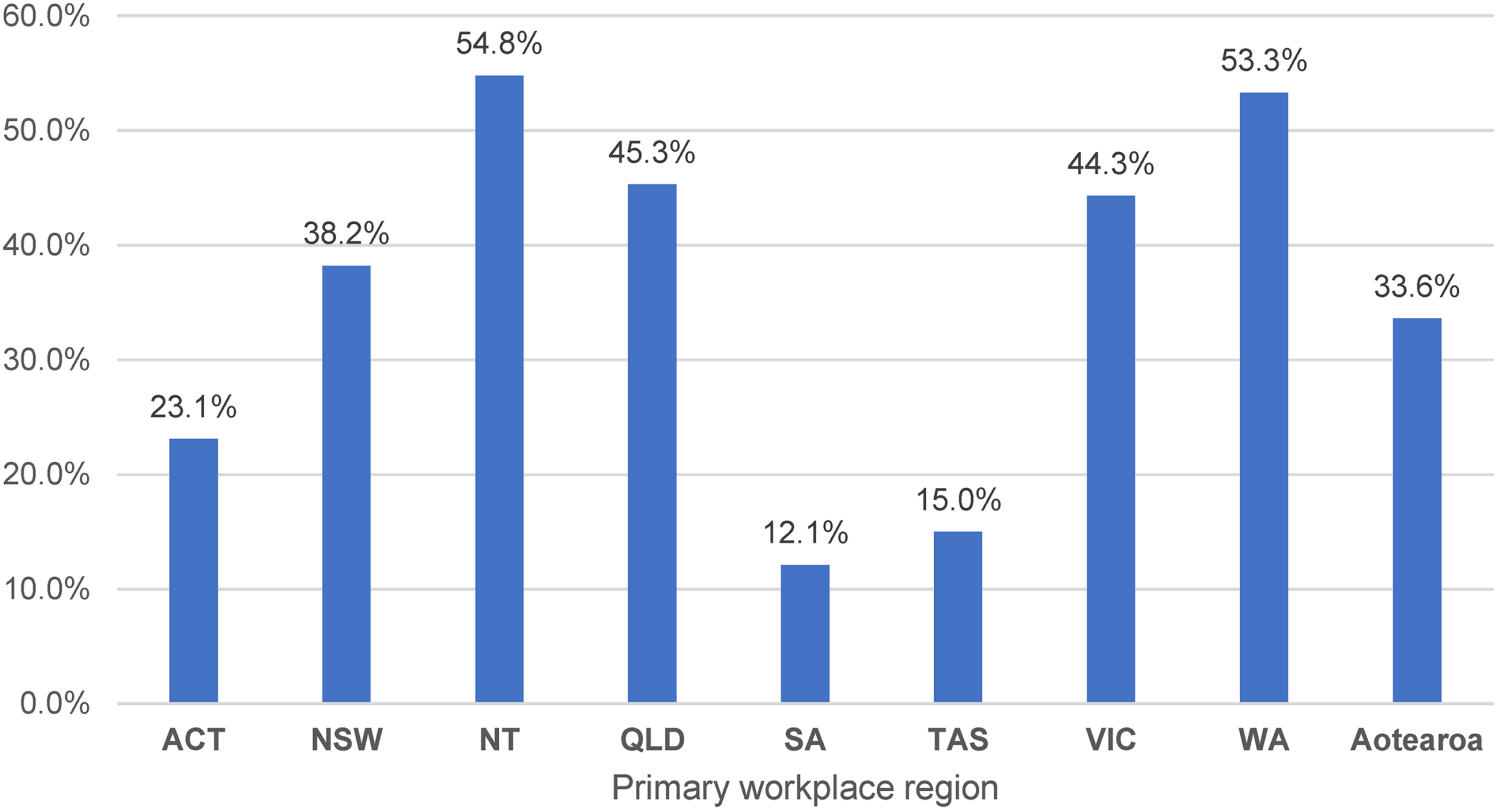
The proportion of respondents reported providing SBIRT for patients at risk of alcohol harm, by region of primary workplace. Error bar shows the lower and upper limit of 95% confidence interval.

Screening, brief intervention and referral to treatment (SBIRT) for patients engaged in risky consumption of alcohol was reported by 39.3% of respondents. Figure 2 demonstrates the breakdown by region.

Alcohol‐related presentations were noted to negatively impact patient waiting times, other patients in the waiting room and care of other ED patients (Table 3). These presentations were also noted to have detrimental effects on staff wellness and job satisfaction (Table 3).

**TABLE 3:** The impact of alcohol‐related presentations on ED function and care of other patients and ED staff, comparing responses between nursing staff and medical doctors

Alcohol‐related presentations impacted aspects of EDs in a negative or very negative way			
	Overall responses, *n* = 1284	Nurses, *n* = 496	Doctors, *n* = 691
	% [95% CI]	% [95% CI]	% [95% CI]
Staff workload	93.1 [91.6–94.4]	96.2 [94.1–97.5]	91.9 [89.6–93.7]
Waiting times	86.1 [84.1–87.9]	87.1 [83.9–89.8]	85.1 [82.2–87.6]
Other patients in the waiting room	94.6 [93.2–95.7]	97.0 [95.1–98.2]	93.1 [90.7–94.7]
The care of other ED patients	87.5 [85.5–89.2]	88.1 [85.0–90.7]	88.1 [85.5–90.3]
Staff wellness	82.4 [80.2–84.4]	83.1 [79.5–86.1]	82.8 [79.8–85.4]
Staff job satisfaction	78.9 [76.6–81.0]	79.6 [75.9–83.0]	79.0 [75.8–81.9]

A total of 728 respondents provided additional free‐text qualitative comments. Box 1 provides some examples of responses.

BOX 1 **Examples of comments on the impacts of alcohol‐related presentations on patient care and ED functions**

Selected qualitative responses to the survey:

The impact of alcohol‐related presentations on patient care

- Someone who is affected by alcohol needs time to sober up. This can delay discharge times and impact on the flow of the ED and seeing other patients who are critically unwell and are at an increased risk of deteriorating in the waiting room. [ED nurse, female]
- Those affected by alcohol in ED often try to interact with other patients, which can lead to aggression in the waiting room or simply making people uncomfortable, causing people greater stress in an already stressful environment [ED clerical staff, self‐specified gender]
- With drunk presentations being a real threat and disruption to others with verbal and (risk of) physical aggression. I think waiting rooms can be a rather horrible place to be, especially for people who feel vulnerable for themselves or their children. [EM specialist, female]
- Elderly patients are 2 m away from an abusive intoxicated patient swearing profanity, unwell patients are waiting for beds that a sedated intoxicated suicidal patient or alcohol withdrawing patient occupies, staff are resigning from the stress of the job leaving us understaffed. [ED registrar, female]

The impact of alcohol‐related presentations on ED function and staff well‐being

- Unpleasant working environment as these patients are commonly abusive to staff. Slows day to day functioning as it takes multiple staff to deal with one heavily intoxicated patient. I often do more work as the registrar doctor to protect the residents and nursing staff (especially junior) from being exposed to their violence. [ED registrar, female]
- [Intoxicated patient] scares children in waiting room. physical violence against other patients – one punched an elderly lady. [ED physician, male]
- I feel we are letting people down badly in this space. We talk about aggression and violence with this group of patients and carers, but there is also a lot of sexual harassment with this group – for example, only last week a patient got very touchy feely with me and he has tracked me down to contact me via social media too. [ED physician, female]
- A drunk father yelling abuse at me while I was trying to care for his child. [Nurse, female]
- It makes me dread working a night shift over the weekend, because that is when we see the effects of alcohol the most – especially in our ED where you are often the only triage nurse on a night shift and you have to deal with managing all the intoxicated patients, and sometimes they make me feel unsafe when taking patients through to the triage bays to be triaged alone. [ED nurse, female]

## Discussion

Almost all ED staff who responded to the survey (97.9%) have experienced alcohol‐related verbal aggression each year and a large majority (92.7%) had been exposed to physical aggression. While numbers are comparable to the survey conducted in 2014,^7^ more than two thirds of respondents believe alcohol related OVA has worsened over the preceding 5 years. Just under half of respondents' experience alcohol related physical violence frequency (weekly) or often (few times per month). ED staff also commonly experience alcohol related OVA from patient's carers or relatives.

Alcohol related presentations have negative effects on overall staff well‐being, ED function and the care of other patients.^8^, ^14^ Adverse working conditions are known to be linked to significant adverse physical and psychological health outcomes and reduce staff retention.^15^ At a time of an international shortage of staff, the survey highlights the urgent need for evidenced based interventions by policy makers for ED settings to mitigate alcohol related aggression and violence toward staff by patients and their carers.

The impact of the COVID‐19 pandemic on alcohol related OVA was less clear with under half of respondents reporting it had worsened. A study in the trend of OVA against ED healthcare staff during the pandemic revealed that there was a significant increase compared to pre‐pandemic rates from an average of 0.05 attacks per 1000/month to 27.2 attacks per 1000/month.^16^ Self‐reported alcohol consumption rates during the pandemic are variable across the population, with socioeconomic factors having an influence on consumption behaviours including stockpiling of alcohol.^17^ The closure of pubs and clubs and a rise in spending on alcohol^11^ may have played a role in the perceived changes in alcohol‐related OVA. In addition, the pandemic had a significant effect on alcohol counselling and treatment services with decreased bed capacity at rehabilitation sites, increased wait times and reduced intake of new clients to rehabilitation and withdrawal services.^18^ Regional data also demonstrates some state‐by‐state variation. Victoria is an outlier in the data compared to other regions. It is the only state where a majority of respondents reported that COVID‐19 had worsened alcohol‐related OVA with figures significantly higher than other regions including South Australia and Northern Territory. This variation may be attributed to the intensity of COVID‐19 restrictions, with Victoria notably experiencing the most lockdowns. In addition, state‐specific differences in alcohol policies could have played a role, for example, Tasmania's increased funding of alcohol and drug services during the pandemic.^19^

It is concerning that since the 2014 survey, ED staff's perception of the incidence of violence has worsened. In Victoria in 2015, an additional funding of $20 million was spent to address the issue of occupational violence in healthcare.^20^ This funding was allocated to implementation of security measures including surveillance cameras, redesigning waiting rooms and alarms. A second round of funding in 2017, reportedly supported the implementation of behaviour assessment rooms (BARs) and increased security personnel. There is evidence to suggest that BARs decrease time in the waiting room as well as the use of restraints, however, require adequate staff to manage patients in these areas.

Not all EDs in Australia and Aotearoa New Zealand have 24/7 trained security staff to protect staff and act as a deterrent.^21^ Given the frequency of alcohol related OVA experienced by ED staff, this is essential. As the frequency of OVA increases, so too will the need for increased security staff, unless effective public health measures are instituted at a population level.

The Violence in Healthcare Taskforce established in 2015 identified a number of key areas to focus on.^22^ Under‐reporting of OVA is a significant issue, perpetuated by workplace culture and attitudes. To combat this, the taskforce encourages implementation of simplified reporting systems to help facilitate reporting. In addition, it advocates for increased education of staff members as well as increasing public awareness through consistent messaging. These are essential measures in contributing to a wider cultural shift when it comes to curbing the use of alcohol in the community and reducing alcohol‐related OVA.

Alcohol‐related harm and the subsequent OVA experienced by ED staff is unacceptable and preventable. The World Health Organization (WHO) recommends there is a role for brief interventions in the ED setting.^23^ The ACEM statement on alcohol harm^24^ also supports preventative strategies such as the use of SBIRT in ED. Completion of an SBIRT for harmful drinking was reported to be performed by less than half the respondents. While research has demonstrated that emergency clinicians support SBIRT but lack time and resources, making this challenging to implement.^25^ Integration of SBIRT into routine ED practice would be a useful, evidence‐based preventative measure to reduce harmful drinking.

The ACEM statement on alcohol harm supports other evidenced‐based policies including pricing, taxation, restricting availability and advertising of alcohol.^17^ This statement also highlights the importance of routine alcohol harm data collection in the ED, both to quantify the scale of the problem and measure the effect of policy changes, specifically the introduction of alcohol‐related harm data elements to the National Minimum Dataset for National Non‐admitted Patient Emergency Department Care (NNAPEDC) in Australia and the National Non‐admitted Patient Collection in New Zealand.^17^ Collecting ‘last drinks’ data in the ED, interagency data sharing and implementing local community‐based interventions, have been shown to reduce the rate of alcohol‐related injury presentations and assaults.^26^, ^27^, ^28^ Its impact on staff OVA has not been studied but the reduction of alcohol‐related injuries is likely to be associated with a reduced assault risk.

### Limitations

Selection and non‐response bias may have occurred because of the voluntary nature of the survey used in the present study. Some misclassification bias may have been introduced by respondence confusing the effects psychotropic drug or medical conditions. The survey focussed on a period of 12 months, which may have introduced a degree of recall bias. Given the very high rates of alcohol related OVA in our 2014 survey, this survey had limited opportunity to demonstrate any increase.

## Conclusion

Alcohol‐related occupational violence and aggression is experienced by almost all ED staff in Australia and Aotearoa New Zealand and is perceived to be increasing. It negatively impacts staff safety, well‐being and retention, the care of other patients and ED function. Stronger evidenced based public policy measures such as price and availability are urgently needed to address in addition to environmental and security.

## Supporting information


**Appendix S1.** Emergency department staff experiences of alcohol‐related presentations in Australia and Aotearoa New Zealand.

**Appendix S2.** Figure data.

## Data Availability

The data that support the findings of the present study are available from the corresponding author upon reasonable request.
