# Supplementary material for: Enough is enough: Alcohol‐related occupational violence and aggression in emergency departments in Australia and New Zealand
Source: Emerg Med Australas. 2025 Mar 7;37(2):e70021. doi: 10.1111/1742-6723.70021 (PMC11886477; doi:10.1111/1742-6723.70021)
Supplement: Supplementary file 1 — Appendix S1. Emergency department staff experiences of alcohol‐related presentations in Australia and Aotearoa New Zealand. [file EMM-37-0-s002.rtf]

Appendix 1: Emergency Department Staff Experiences of Alcohol-related Presentations in Australia and Aotearoa New Zealand


The aim of this survey is to investigate your experiences of alcohol-related presentations at your emergency department (ED).

By alcohol-related presentations, we mean all presentations relating to alcohol, including acute alcohol poisoning, alcohol withdrawal, alcohol-related accidents/injuries in the home and workplace, motor vehicle accidents, self-injury and assault.

The survey questions mirror the Australasian College for Emergency Medicine (ACEM) research undertaken in 2014 and so the findings will be compared to the 2014 survey findings to re-examine the scale of adverse impacts of alcohol on ED staff and patient safety and to identify changes over time. This will also provide benchmark data from ED staff across Australia and Aotearoa New Zealand on their experiences of the impact of alcohol-related presentations on ED staff and ED function.

Your responses are anonymous, and the information collected will be strictly confidential. No personal or hospital specific data will be collected. The survey should take about 10 minutes to complete. At the end of the survey, we have included some open-ended questions if you would like to provide more information. After you have submitted the survey you will be asked if you are interested in being part of a media/ advocacy campaign on alcohol-related ED presentations. If you are interested, you can choose to share your email address, it will be uploaded separately from your survey responses to ensure your survey responses are not linked to your contact information.

The survey results will be used promote community discussion and to deliver a media advocacy campaign on the impact of alcohol-related harm on EDs across Australia and Aotearoa New Zealand. Findings will be made available on the ACEM website and published in emergency medicine scientific journals and/or presented at scientific conferences, as well as through the media.

Ethics approval for this survey has been given by Monash Health Human Research Ethics Committee. ACEM has received funding from the Australian Rechabite Foundation for this project. 

The College of Emergency Nursing Australasia (CENA) is generously supporting ACEM in this research.If you have questions about the survey you can contact the ACEM's Research Manager, Jolene Lim at Jolene.Lim@acem.org.au or call +61 (03) 9320 0471. If you have any complaints, or concerns about the way the research is being conducted, you can contact Monash Health Human Research Ethics Committee (HREC) Executive Officer at research@monashhealth.org or call +61 (03) 9594 4611.

Thank you very much for your time and support. Please start by clicking on the Start button below.


Are you currently working or have you worked in an ED in Australia or Aotearoa New Zealand in the previous 12 months?
1.	Yes
2.	No


Section A: Demographics

In which region do you primarily work in?
1.	ACT
2.	NSW
3.	NT
4.	QLD
5.	SA
6.	TAS
7.	VIC
8.	WA
9.	Aotearoa New Zealand

Is your primary hospital:
1.	Public
2.	Private

Is your ED located in a:
1.	Major city location
2.	Regional location
3.	Remote location

What is your ED role?
1.	Alcohol and other drug support worker
2.	Cleaner
3.	Clerical staff/ administrator
4.	Indigenous health worker
5.	Nurse
6.	Nurse practitioner
7.	Physician/ Specialist
8.	Registrar
9.	Other Medical Officer
10.	Medical Intern
11.	Mental health worker
12.	Peer support worker
13.	Occupational therapist
14.	Patient service assistant/ orderly
15.	Pharmacist
16.	Physiotherapist
17.	Radiologist
18.	Security guard
19.	Social worker
20.	Other (please describe) __________

What is your gender?
1.	Male
2.	Female
3.	Prefer to self-describe
4.	Prefer not to say


How many year(s) of working in emergency departments do you have?
1.	&lt; 1 year
2.	1 – 5 years
3.	6 – 10 years
4.	11 – 20 years
5.	21 – 30 years
6.	> 30 years


Section B: Your perceptions of how alcohol-related presentations affect aspects of your ED

How do you think alcohol-related presentations affect the following in your ED:

	In a very positive way	In a positive way	Neutral	In a negative way	In a very negative way	Don't know/ Not applicable	
Staff workload	❏	❏	❏	❏	❏	❏	
Waiting times	❏	❏	❏	❏	❏	❏	
Other patients in the waiting room	❏	❏	❏	❏	❏	❏	
The care of other patients in the ED	❏	❏	❏	❏	❏	❏	
Staff wellness	❏	❏	❏	❏	❏	❏	
Staff job satisfaction	❏	❏	❏	❏	❏	❏	

ED staff at my hospital need more training to respond to alcohol-related presentations
1.	Strongly disagree
2.	Disagree
3.	Neither agree nor disagree
4.	Agree
5.	Strongly agree
6.	Don&#39;t know/ Not applicable

If you have comments or anecdotes relating to this section, please write them here:


	


Section C: Your experiences of alcohol-related violence in the ED

This section asks you about any alcohol-related workplace aggression you have experienced in the last 12 months.By aggression we mean:Verbal or written abuse, threats, intimidation or harassment - such as ridicule, abusive email, racism, bullying, contemptuous treatment and non-physical threats or intimidationPhysical threats, intimidation, harassment or violence - such as raised hand or object, unwanted touching, damage to property and sexual or other physical assault

Have you ever felt unsafe due to the presence of an alcohol-affected patient while working in your ED?
1.	Yes
2.	No


For each potential source of alcohol-related aggression, please select the option that most closely matches how often you have experienced each type of aggression in the last 12 months:

	Frequently (once or more per week)	Often (a few times each month)	Occasionally (a few times each 6 months)	Infrequently (a few times in 12 months)	Not at all	
Alcohol-related aggression from patients: Verbal or written abuse, threats, intimidation or harassment	❏	❏	❏	❏	❏	
Alcohol-related aggression from patients: Physical threats, intimidation, harassment or violence	❏	❏	❏	❏	❏	
Alcohol-related aggression from relatives or carers of patients: Verbal or written abuse, threats, intimidation or harassment	❏	❏	❏	❏	❏	
Alcohol-related aggression from relatives or carers of patients: Physical threats, intimidation, harassment or violence	❏	❏	❏	❏	❏	

How has COVID impacted the incidents of alcohol-related violence in the ED?
1.	A lot worse
2.	A little worse
3.	No change
4.	A little better
5.	A lot better

How have the incidents of alcohol-related violence changed in your ED over the past five years?
1.	A lot worse
2.	A little worse
3.	No change
4.	A little better
5.	A lot better

If you have comments or anecdotes on this section, please write them here:


	


Section D: Screening and brief interventions in your ED

Does your ED routinely screen patients for the consumption of alcohol?
1.	Yes
2.	No

Does your ED provide Screening, Brief Intervention, and Referral to Treatment (SBIRT) for patients at risk of alcohol harm?
1.	Yes
2.	No


What alcohol-related screening questions are used in your ED:


	


What alcohol-related brief interventions are used in your ED:


	


Section E: Additional Questions

If you have time, we'd love to hear back from you on the following questions. If not, please skip the following questions and finish the survey by clicking on the submit button at the bottom.

How does seeing alcohol-related presentations to the ED make you feel about your job?


	


What effect do alcohol-related presentations have on other patients attending your ED?


	


What effect do you think alcohol-related presentations have on the day-to-day functioning of your ED?


	


What do you think could be done to reduce alcohol-related presentations to your ED?


	


If you have any other comments or anecdotes relating to this section, please write them here:


	
